# Impacts of Clarification Techniques on Sample Constituents and Pathogen Retention

**DOI:** 10.3390/foods8120636

**Published:** 2019-12-03

**Authors:** Cheryl M. Armstrong, Andrew G. Gehring, George C. Paoli, Chin-Yi Chen, Yiping He, Joseph A. Capobianco

**Affiliations:** United States Department of Agriculture, Agriculture Research Service, Eastern Regional Research Center, 600 East Mermaid Lane, Wyndmoor, PA 19038, USA

**Keywords:** filtration, foodborne pathogen, graphite felt, glass wool, polypropylene filter, continuous flow centrifugation, detection

## Abstract

Determination of the microbial content in foods is important, not only for safe consumption, but also for food quality, value, and yield. A variety of molecular techniques are currently available for both identification and quantification of microbial content within samples; however, their success is often contingent upon proper sample preparation when the subject of investigation is a complex mixture of components such as foods. Because of the importance of sample preparation, the present study employs a systematic approach to compare the effects of four different separation techniques (glass wool, 50 μm polypropylene filters, graphite felt, and continuous flow centrifugation (CFC)) on sample preparation. To define the physical effects associated with the use of these separation methods, a multifactorial analysis was performed where particle size and composition, both pre- and post- processing, were analyzed for four different food matrices including lean ground beef, ground pork, ground turkey and spinach. Retention of three important foodborne bacterial pathogens (*Escherichia coli* O157:H7, *Salmonella enterica*, and *Listeria monocytogenes*) was also examined to evaluate the feasibility of the aforementioned methods to be utilized within the context of foodborne pathogen detection. Data from the multifactorial analysis not only delineated the particle size ranges but also defined the unique compositional profiles and quantified the bacterial retention. The three filtration membranes allowed for the passage of bacteria with minimal loss while CFC concentrated the inoculated bacteria. In addition, the deposition and therefore concentration of food matrix observed with CFC was considerably higher for meat samples relative to spinach. However, filtration with glass wool prior to CFC helped clarify meat samples, which led to considerably lower amounts of solids in the CFC vessel post processing and an increase in the recovery of the bacteria. Overall, by laying a framework for the deductive selection of sample preparation techniques, the results of the study can be applied to a range of applications where it would be beneficial to scientifically guide the pairing of the criteria associated with a downstream detection method with the most advantageous sample preparation techniques for complex matrices such as foods.

## 1. Introduction

The microbial content in foods is not only important for safe consumption, but also affects the quality, value, and yield for some production processes (e.g., fermentation), along with affecting the ability to avert negative economic impacts attributable to premature spoilage. The presence of unwanted bacteria may simply affect the organoleptic properties of foods or may shorten the shelf-life resulting in potential waste and lost revenue. Contaminating bacteria may also be responsible for foodborne illnesses, resulting in unwanted medical expenses and time off from work, chronic sequelae or even death [1,2].

Rapid methods that can both detect the presence of and identify pathogenic bacteria, which are sometimes only found at very low levels, are highly desirable [3,4,5]. Because these methods may be used for on-line or near-line microbiological screening, food production may proceed unimpeded. In general, use of these methods allow for the timely certification of any product held back from release due to testing.

In the analysis of foods for the presence of contaminating bacteria, representative samples frequently benefit from the application of sample pretreatment. Common practices can include adjustment of pH, ionic strength, and separation-based techniques employed to remove bulk food matrix and potential inhibitors that may confound downstream analysis. Problems that may arise when emerging detection technologies are applied to food include fouling of membrane surfaces used for visual analysis as with microscopic observation [6,7] or laser-based scanning of fluorescently labeled and membrane-captured cells [8]; and clogging of microfluidic analysis device flow channels [9,10,11]. Other sample matrix related problems may arise from particle-based steric hindrance and/or occlusion of optical techniques (e.g., microscopy and colorimetric or UV-Vis spectroscopic approaches), non-specific binding, or unwanted cross-reactions involving background microbes or sample matrix, etc. that may yield false-positive or -negative results for immunoassay. The presence of inhibitors in food matrices may also adversely affect molecular detection methods such as polymerase chain reaction (PCR) or loop-mediated isothermal amplification (LAMP) [12]. Examples of separation techniques that can mitigate such effects include centrifugation [13,14], Percoll [15], prefiltration/filtration [16,17,18], affinity chromatography [16,18,19,20,21], sedimentation [22,23], magnetic ionic liquids [24], surface acoustic wave [25], and particle-based separation [26,27].

Methods intended to separate target bacteria from the bulk food matrix can also affect the concentration of bacteria within the sample. For example, centrifugation can increase the concentration of bacteria within a sample prior to analysis. This can be a desirable outcome as it could improve the limit of detection of downstream assays intended to detect zero tolerance organisms. However, it can be undesirable for systems with limited dynamic range. Separation can also reduce the concentration of bacteria, leading to unintended consequences such as false negative detection outcomes. Thus, for size exclusion and preferential adsorption/absorption techniques, constraints on pore size and material composition limit the ability to effectively separate components from one another. As such, the requirements of downstream detection schemes can place fundamental restrictions on the separation technique.

The design of the experimental protocols used in this investigation align with sampling methodologies and detection assays adopted by regulatory agencies in the United States. The present work investigated the composition of commonly tested foods including ground meats (beef, pork, and turkey) and a leafy vegetable (spinach) before and after application of a separation method. The effects of the different separation approaches on particle size distribution were also studied. In addition, food homogenates were inoculated with important foodborne pathogens (*Escherichia coli* O157:H7, *Salmonella enterica*, and *Listeria monocytogenes*) and bacterial recovery was assessed for the different foods and separation conditions tested. 

## 2. Materials and Methods

### 2.1. Food Matrix Preparation

The experiment was designed to keep the volume of food homogenate constant at ~1 L, while retaining the specified food dilution ratios, 1:4 (meat samples) and 1:10 (spinach), as specified in the United States Department of Agriculture (USDA) Microbiological Laboratory Guidebook (MLG) [28] and United States Food and Drug Administration (FDA) Bacteriological Analytical Manual (BAM) [29], respectively. Ground beef (90% lean/10% fat), ground pork, or ground turkey (93% lean/7% fat) purchased from a local grocery retailor were partitioned into 325 g samples and placed into a 4080 mL (15 × 15 inch) sterile Stomacher bag containing a finely perforated polyethylene membrane filter with a pore diameter of 330 µm (Whirl-Pak cat# B01525, Nasco, Fort Atkinson, WI, USA) using sterile utensils per the USDA MLG sampling procedure with modification [28]. Spinach, partitioned into 111 g samples, was placed into Stomacher bags identical to that used for the meats. One liter of sterile Luria-Bertani (LB) broth was added to each filtered Stomacher bag containing sample before being homogenized in the Seward Stomacher 3500 (Seward Laboratory Systems Inc., Islandia, NY, USA ) for 30 s at 150 rpm. After homogenization, the liquid was removed from the outer portion of the membrane filter within the Stomacher bag for further processing as described below. When more than 1 L was necessary, samples were processed 1 L at a time and pooled in a sterile glass Erlenmeyer flask with stirring using a magnetic stir bar to help provide sample consistency. Samples labeled as “stomacher bag” did not undergo any additional processing, while the remaining samples were further processed using one of following methods.

#### 2.1.1. Glass Wool (GW) Filtration

The glass wool filters were prepared by removing the existing 0.45 µm filter from a Corning 1 L Sterile filter system (cat#430516; Corning, NY, USA) and replacing it with approximately 10 grams of glass wool (VWR International, Radnor, PA, USA) to cover the base of the funnel. Samples were allowed to pass through the filter using gravity flow initially. When the gravity flow was no longer a continuous stream, vacuum was applied via an in-house vacuum line. If the membrane became sufficiently clogged so that the vacuum applied could not pull the residual liquid through, the remaining material was transferred to a second GW filter. Then, gravity flow and vacuum were once again applied as described above. It is important to note that the spinach samples only required the use of a single filter because no clogging occurred.

#### 2.1.2. 50 Micron Polypropylene Filter (50 μm filter)

The 50 μm filters were prepared by removing the existing 0.45 µm filter from a Corning 1 L Sterile filter system and replacing it with a square piece (15.5 cm × 13.5 cm) cut from a 50 μm welded polypropylene felt liquid bag filter with a plastic flange (AJR Filtration, St. Charles, IL, USA). Filters were glued to the base of the funnel using a glue gun to ensure the sample flowed through the filter as opposed to around it. Both gravity flow and an in-house vacuum were applied as described above for the glass wool filtration.

#### 2.1.3. Graphite Felt (GF) Filtration

The graphite felt filters were prepared by removing the existing 0.45 µm filter from a Corning 1 L Sterile filter system and replacing it with a square piece (15.5 cm × 13.5 cm) of graphite felt (Electrosynthesis, Lancaster, NY, USA), which was glued to the base of the funnel using a glue gun. Both gravity flow and an in-house vacuum were applied as described above for the glass wool filtration.

#### 2.1.4. Continuous Flow Centrifugation (CFC)

**Figure 1:**
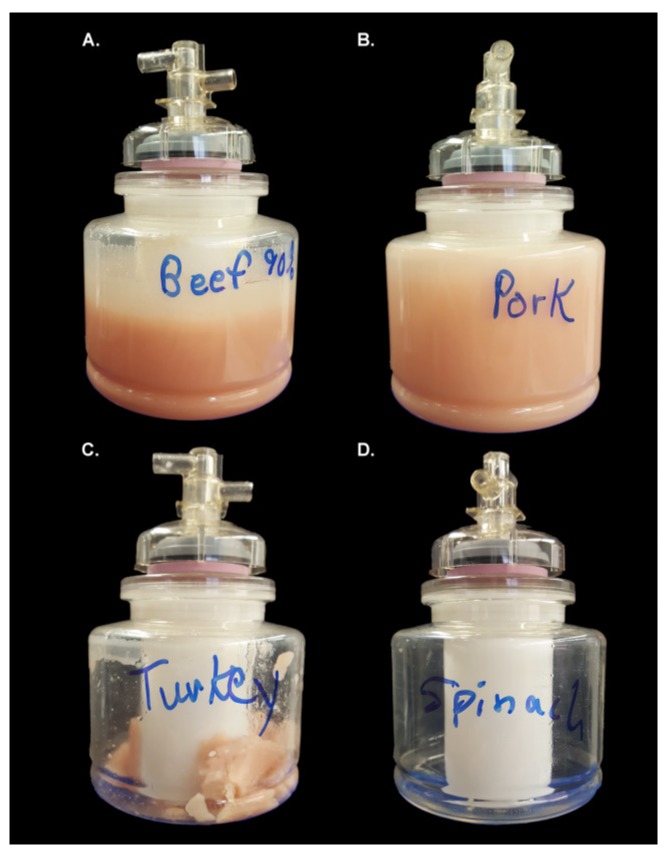
Deposit formation resulted from continuous flow centrifugation. Photographs of CFC bowls after sample processing. Deposits accumulated along the inside wall of the CFC bowl when processing (**A**) ground beef, (**B**) pork, and (**C**) turkey, but not (**D**) spinach.

Continuous flow centrifugation was performed on samples previously processed via the Stomacher using the CFC Express (Scientific Methods, Granger, IN, USA). For the CFC, a Masterflex pump set at speed 3 (~60 mL/min) and Masterflex L/S platinum-cured silicone tubing aided in the transfer of the sample into the disposable bowl of the CFC. Samples were spun at 10,000 rpm for ~15 min, which concentrated the sample to a reduced volume of ~250 mL. After centrifugation, the liquid retentate was subsequently poured from the CFC bowl to a sterile Corning 250 mL bottle for storage without disturbing any sample material that had been pressed against the side of the CFC bowl (referred to herein as the solid retentate) (Figure 1). Effluents, defined as the liquid which passed through the CFC bowl and exited through the outlet port, were also collected for analysis.

### 2.2. Particle Size Determination

Dynamic light scattering (DLS) was used to investigate the effect of the filtration treatment on the samples. The DLS measurements were taken using a Mastersizer 3000 (Malvern Instruments; Worcestershire, UK) equipped with two light sources (red (λ = 633 nm) and blue (λ = 470 nm)) and Malvern’s small volume sample dispersion unit (SVDU). The following constant values were selected from a database contained in the software provided by Malvern Instruments or from the literature for the respective samples: the refractive index of the meats was 1.345 [30,31] and the density of the meats was 1.05 g/cc [32]; the refractive index of the spinach was 1.45 (Malvern technical support personal communication and [33]) and the density of the spinach was 0.08 g/cc [34]; and the refractive index of the solvent (water) was 1.33 and the density of the water was 1.0 g/cc. Nanopure water was prepared using a Barnstead ultrapure water purification system (Thermo Fisher Scientific, Inc., Canoga Park, CA, USA).

Prior to sample readings, 130 mL of nanopure water was added to the SVDU and circulated through the system using a stirring rate of 1500 rpm. The laser system was aligned and a background measurement was collected using a 10-s duration for both blue- and red-light conditions. A vortex mixer at maximum speed agitated the samples for 90 s prior to the measurements being taken to resuspend any precipitated material. In order to ensure the obscuration values were in the range suggested by the manufacturer (5–10%), 200 µL of the meat/spinach suspension was added to 130 mL of nanopure water using wide orifice pipette tips. Using methods similar to those employed for the background measurements, the suspension was recirculated through the system using a stirring rate of 1500 rpm, and the sample measurements were collected using a 10-s duration for both blue- and red-light conditions. The number of measurements collected for each sample was set to 10 with a 1-s delay between measurement cycles. General purpose analysis for non-spherical particles (Mie theory) generated a volume-based size frequency distribution for particle sizes ranging from 10 nm to 3500 µm. The Mastersizer 3000 also reported the concentration on a per volume basis. 

The unit was cleaned following each experimental sample measurement. The cleaning cycle consisted of circulating 130 mL of nanopure water through the system for 2 min, and then completely draining the rinse water from the system. The process was repeated three times and the background measurement collected using the 130 mL of nanopure water retained in the system during the final cleaning cycle. 

### 2.3. Sample Composition Determination

The amount of (1) protein, (2) fat, (3) moisture, (4) ash, and (5) total carbohydrates within each sample was determined via nutritional analysis. Samples were prepared in-house and ~250 mL of each of the samples were shipped on ice overnight to Société Générale de Surveillance (SGS Brookings, SD, USA) for sample analysis. Samples were measured using the following accredited methods: (1) crude protein was analyzed via ANA030CHEM; (2) total, saturated, and unsaturated fats were analyzed using gas chromatograph- flame ionization detector via Association of Official Agricultural Chemists (AOAC) International Official Methods of Analysis (OMA) 996.06; (3) moisture content was measured via ANA035CHEM; (4) crude ash content was measured using AOAC 942.05 and (5) carbohydrate content was calculated via the Code of Federal Regulations (CFR)21 method.

### 2.4. Bacterial Strains and Growth Conditions

Selection of the strains of *Escherichia coli* O157:H7, *Salmonella enterica*, and *Listeria monocytogenes* used in this study was based on the antibiotic resistances that would allow the strains to be individually enumerated when the three pathogens were mixed with the natural microbiota from foods. *Escherichia coli* O157:H7 PC is a spectinomycin-resistant derivative of the Shiga toxin negative strain ATCC 43888 [35], *Salmonella enterica* subsp. *enterica* serovar Minnesota strain K^+^ is a kanamycin-resistant derivative of *S.* Minnesota (Paoli and Uhlich, unpublished data) and *Listeria monocytogenes* 10403S is a streptomycin-resistant isolate of strain 10403 [36]. The *E. coli* O157:H7 PC and *S.* Minnesota K^+^ strains were routinely plated on LB agar containing 400 µg/mL spectinomycin and 50 µg/mL kanamycin, respectively. *L. monocytogenes* 10403S was plated on Brain Heart Infusion (BHI) media containing 1 mg/mL streptomycin. When grown on plates, the media was solidified with 1.5% agar. Both plates and liquid cultures were incubated at 37 °C. The concentrations of antibiotics used were determined empirically for each strain such that they allowed for the selection of only one targeted pathogen (i.e., each pathogen was able to grow on only one of the antibiotic-containing selection plates, with its growth being inhibited on plates containing either of the other two antibiotics at the selected concentrations).In addition, prior to carrying out experiments in inoculated foods, ground beef and chicken homogenates were plated on each of the aforementioned antibiotic-containing media, to help ensure that growth of the natural microbiota would not interfere with pathogen enumeration. 

### 2.5. Preparation of Inoculum and Filtering of Spiked Samples

Artificially contaminated food samples were prepared in order to simulate food homogenates, post enrichment in a manner that ensured that the number of pathogens was relatively consistent between trials. Single colony isolates of each bacterial strain (*E. coli* O157:H7 PC, *S.* Minnesota K^+^, and *L. monocytogenes* 10403S) were picked from agar plates and inoculated into separate 5 mL tubes containing LB or BHI with the appropriate antibiotics and grown overnight (~18 h) at 37 °C with shaking (180 rpm). Then, each culture was adjusted to an OD_600_ of 1.0 (~10^9^ CFU/mL) with fresh media. The three cultures were individually enumerated using the 6 × 6 drop plate method [37] on plates containing the appropriate antibiotics. The prepared inoculum of each pathogen was added to stomached food homogenate at a ratio of 1:1000 (e.g., 2 mL of each of the three pathogen inocula were added to 2 L of food homogenate). A 333 mL aliquot of each inoculated stomached food homogenates (beef, pork, turkey, and spinach) was filtered using the filtration process described above for the corresponding filtration devices (GW, 50 µm filter, and GF) with the exception of the CFC, for which a 1 L volume of each inoculated stomached food homogenate had to be used because of the collection bowl size restriction of the CFC machine. Using this method, approximately 300 mL was collected post-filtration in a sterile Corning 1 L storage bottle. A 1.5 mL sub-sample from each stomached and filtered food matrix containing pathogens was enumerated using the 6 × 6 drop plate method. For thoroughness, enumeration was performed on samples taken from uninoculated foods as well as the CFC effluent were enumerated. Three independent replicates were performed for each of the separation methods and food matrices reported.

### 2.6. Tandem Filtration of Spiked Ground Beef Samples

Bacterial cultures were prepared for use as inoculum as described above. The prepared inocula described above were added to stomached homogenates of lean ground beef at a ratio of 1:1000 (e.g., 2 mL of each of the three pathogen inocula were added to 2 L of food extract). A 1-L aliquot of the inoculated stomached beef homogenates was subjected to either CFC alone, or filtered via GW as described above under “*Glass wool filtration*” and then subjected to CFC (GW + CFC). The resulting liquid retentate was collected post-processing in a sterile Corning 1 L storage bottle with a 1.5 mL sub-sample being removed from both the CFC alone and the GW + CFC filtered food matrix containing pathogens for enumeration. For thoroughness, samples were taken from uninoculated food samples as well as the CFC effluent and immediately after the GW filtration for enumeration. Three independent replicates were performed for each separation technique reported.

### 2.7. Culture Plate Photomicrography

A VHX-5000 (version 1.6.1.0) digital microscope (Keyence Corp., Elmwood Park, NJ, USA) running system software version 1.04 was employed for photomicrographic documentation of 6 × 6 bacterial drop plates under ambient lighting. The following settings were used: culture plates were upright with lids removed, top-down view with 20-degree counterclockwise rotation (−20 tilt angle) of the lens/CMOS, black stage (at lowest height), full coaxial lighting set at maximum intensity of 255, 50× zoom lens (Z00), 5× zoom (X5), high resolution high dynamic range (or HHDR) mode, and captured images saved in tagged image file format. Photomicrographic images were cropped and resized for presentation purposes. 

### 2.8. Data Analsysis

All of the raw data was imported into and analyzed by JMP software version 14 (SAS Institute, Cary, NC, USA)). This software was used to prepare the presented figures and compare data sets using statistical tests. The level of significance was assessed using a type one error (α) of 5%.

## 3. Results

### 3.1. Particle Size Distribution was Affected by Sample Preparation Techniques

The size of the particles within a sample often dictates the ability to implement specific detection devices. For example, if particle sizes are too large microfluidic-based devices clog, rendering them useless. Because knowledge concerning the particle size distribution resulting from the use of a filter can be imperative for the development of applicable/appropriate sample preparation techniques, we determined the size distribution of particles post-treatment for several different separation processes commonly employed for sample clean-up. Here, size distribution of the particles was determined via dynamic light scattering, where sample concentration was measured by obscuration. In theory, scattering data from a diffraction system assumes that the light striking the detector has been scattered by a single particle; therefore, multiple scattering events, and the composite nature of the samples impact the accuracy of the measurements, which must be taken into consideration during this analysis. 

**Figure 2:**
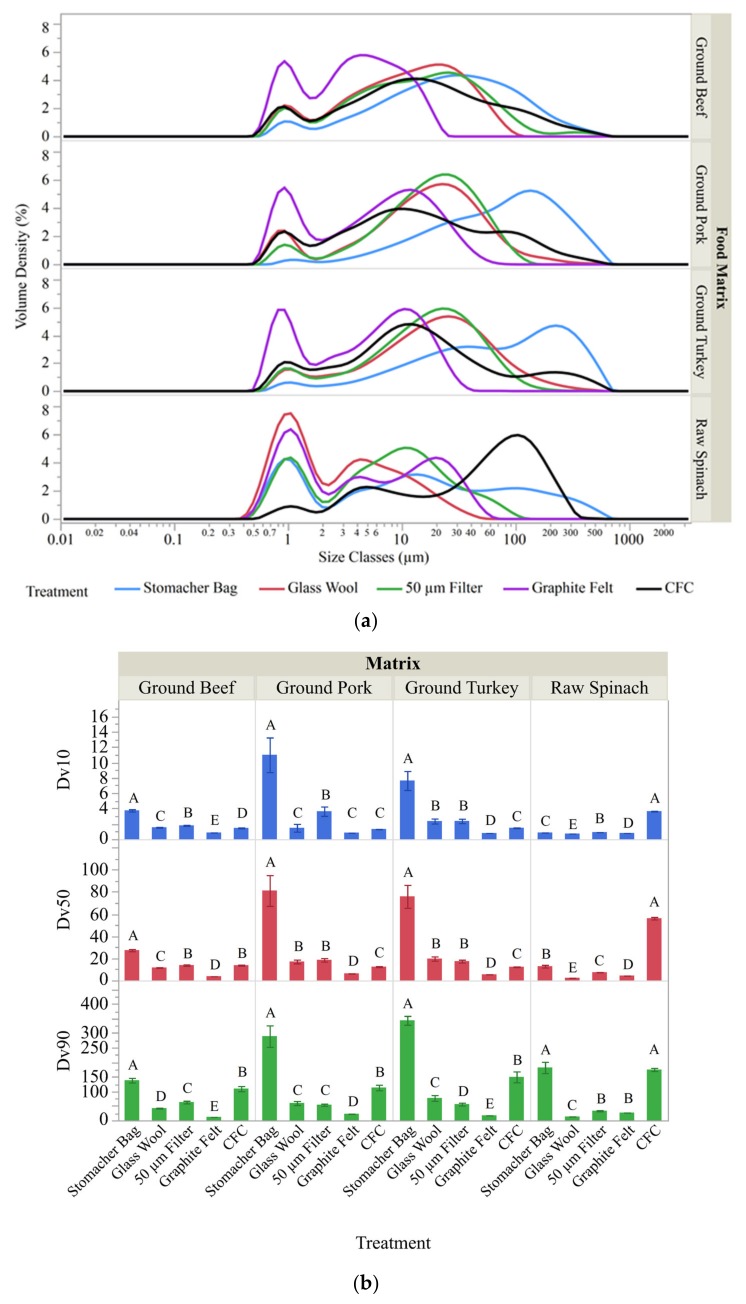
Particle size analysis. (**a**) Particle size distribution was determined with dynamic light scattering. Sample matrices consisted of ground beef, ground pork, ground turkey and spinach as indicated on the right axis. All samples were initially processed via a filtered stomacher bag (blue line) to mix the sample and remove extremely large particulates and is considered the pre-filtration reference point for these experiments. Samples were then subjected to the stated separation methods and the particle size distribution is shown post-processing via glass wool – red line; 50 μm filter – green line; graphite felt – purple line; and continuous flow centrifugation (CFC) – black line. (**b**) Average values collected for treated samples corresponding to the size points in the distribution patterns where 10, 50, or 90% of the total material volume was either equal to or smaller than the listed size (Dv 10, Dv 50, or Dv 90 respectively) are grouped by food matrix. Student’s t-tests were utilized to compare the effect of treatments on the Dv 10, Dv 50 and Dv 90 calculated from particle size distribution data. Statistically different values (*p* < 0.05) are indicated by the connecting letters report. The letters are organized within groups from A-E with the alphabetical progression being associated with lower means.

Overall, data resulting from this analysis demonstrated that a broad range of particle sizes were obtainable depending upon the separation technique utilized (Figure 2a). All sample matrices and separation devices utilized displayed a multimodal distribution pattern, with one of the maxima centered around the 1 μm size range. Sample removed from the stomacher bag without any further separation (Figure 2a, blue line) showed the widest range of particle sizes. This was expected since the screen in the stomacher bag acted as the only separation system for these samples. All other samples were processed though the screen in the stomacher bag, followed by the additional method reported on the graph. It is interesting to note that in many cases the size distribution pattern that resulted from the 50 μm filter closely resembled that of the GW.

For all of the ground meat samples the size classes resulted from each treatment appeared relatively similar for the different food matrices, with the stomacher bag and the CFC yielding particles of the largest size, followed by either the GW or the 50 μm filter (Figure 2b, Dv 90). The GF yielded the smallest particles, where the Dv (90) values were 12.3, 23, and 16.7 for ground beef, pork, and turkey, respectively. The behavior of the spinach, being the only food matrix that consisted of plant material, was unique in the fact that GW yielded a Dv (90) value of 13.7 compared to a Dv (90) value of 27.2 for the GF. 

### 3.2. Sample Preparation Methods Resulted in Compositional Changes to the Matrix

Assay inhibition can also be problematic when attempting to detect foodborne pathogens using molecular methods because of the complexities associated with the matrices in which they are found [38,39]. To identify techniques that may aid in the removal of food-based components acting to inhibit a chosen detection platform, a compositional analysis both before and after sample clarification via GW, a 50 μm filter, GF, or CFC was performed. The compositional analysis established the amount of protein, fat, moisture, ash, and total carbohydrates within each sample. Initially, all samples were homogenized in the Seward Stomacher 3500 using a Whirl-Pak bag containing a polyethylene membrane filter before further clarification was performed. This allowed the stomached sample to act as the pre-processed control.

**Figure 3:**
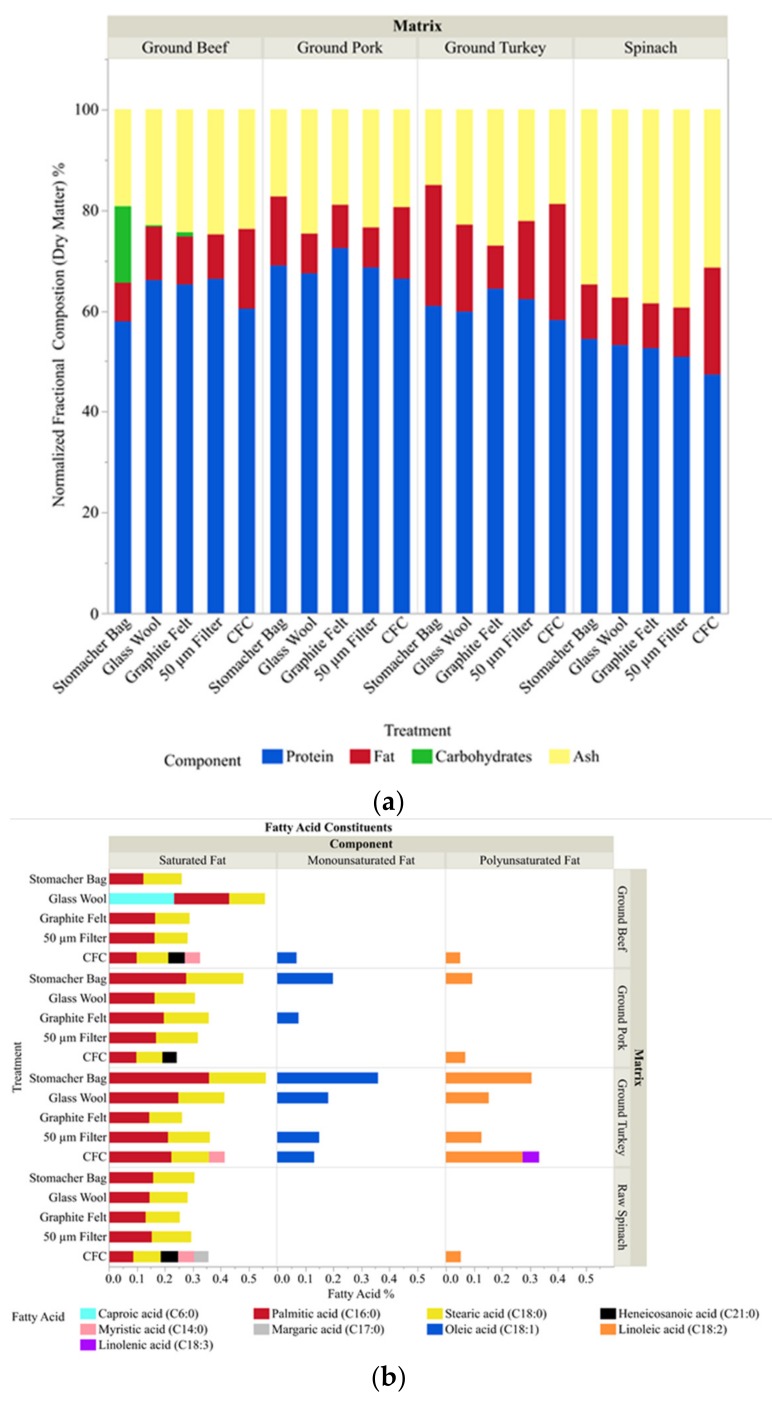
Sample composition. (**a**) The relative amount of protein, carbohydrate, fat, and ash within each sample was determined based on dry matter basis. The data was normalized by dividing each measured (or calculated) dry matter value by the total protein, fat, carbohydrates and ash content. (**b**) Delineation of fats found within the homogenate of the individual samples into their fatty acid constituents.

Each different matrix and separation device utilized created a unique profile, although some general trends did emerge from the data collected. Overall moisture was the majority component of the sample (>95%) and varied less than 3% from sample to sample. Thus, the results were analyzed and are presented in Figure 3a on a dry matter basis. For example, the percent crude ash on dry matter increased for all treatments with the exception of the CFC with spinach as the matrix. (Figure 3a, yellow bar). In addition, the percent fat in dry matter was reduced by the 50 micron filter, GW and GF when spinach, pork or turkey was the matrix (Figure 3a, red bar). This observed reduction in percentage fat is amplified as the level initially present is increased. This is in stark contrast to the CFC, which did not appear to substantially decrease the amount of fat in turkey or pork and even appeared to concentrate the amount of fat in both 90% lean/10% fat ground beef and spinach post-processing. Moreover, an in-depth analysis of the types of fat within the turkey samples revealed the unique ability of the GF to remove monounsaturated and polyunsaturated fats, in particular oleic and linoleic acid (Figure 3b), from the sample matrix.

### 3.3. High Percentage of Bacterial Recovery Post-processing

**Figure 4:**
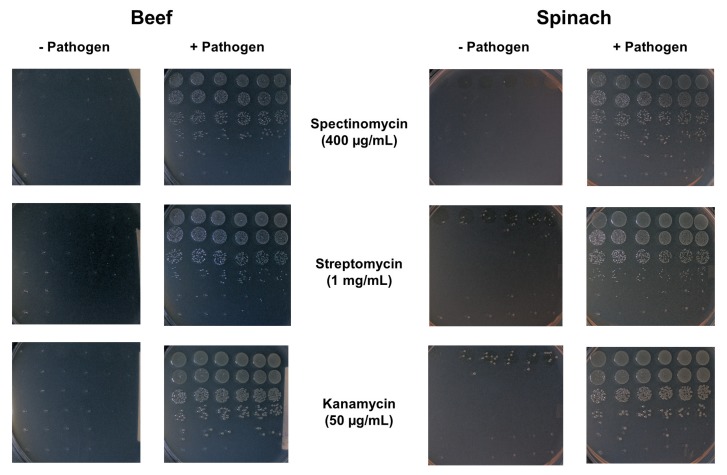
Natural microbiota on antibiotic-containing culture plates. To determine if any of the microbiota from the matrices chosen for testing (ground beef, ground pork, ground turkey and spinach) were sufficiently resistant to the antibiotics (spectinomycin, streptomycin, or kanamycin) at the concentrations used in this study, which may interfere with pathogen enumeration, samples were plated onto agar plates containing each of the three antibiotics and photographed after an overnight incubation at 37 °C. Plates shown are representative of the beef and spinach trials before inoculation (“− Pathogen”) and after the inoculation cocktail was added (“+ Pathogen”).

In order to verify the passage of possible bacterial pathogens that can be found in the different food matrices through the proposed filtration/separation devices, a multifactorial analysis was performed to establish the presence of both Gram positive (*L. monocytogenes*) and Gram negative (*E. coli*, and *S. enterica*) pathogenic bacteria within the filtrate of the five different filtration systems (stomacher bag, GW, 50 μm filter, GF, and CFC) in the presence of four diverse food matrices (ground beef, ground pork, ground turkey, and spinach). For this analysis, a cocktail containing *E. coli*, *L. monocytogenes*, and *S. enterica* was added to the stomached food to a final concentration of ~10^6^ CFU/mL each before each sample was filtered. Filtrates were then collected and 6 × 6 drop plating was performed on agar plates containing an antibiotic for selection of the respective bacteria. Because the presence of antibiotic resistant organisms in the food microbiota could potentially interfere with pathogen enumeration, the presence of antibiotic resistant bacteria present in the initial food matrices was investigated by plating a stomached sample of each of the four food matrices (prior to the addition of the inoculum) on agar plates containing the antibiotics (Figure 4).

**Figure 5:**
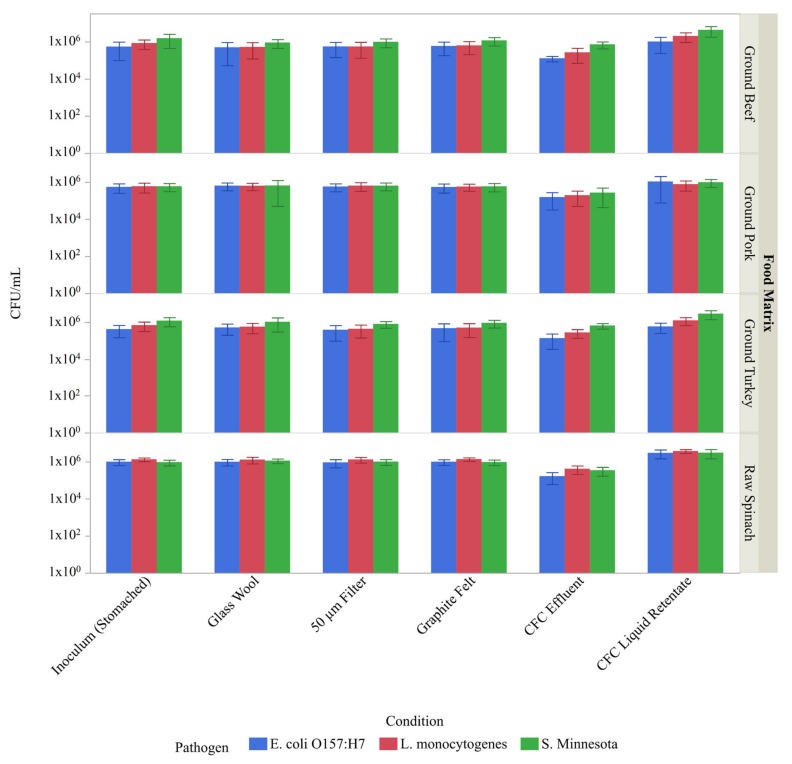
Microbial cell count in filtrates. Samples consisting of A) ground beef, B) ground pork, C) ground turkey, and D) spinach were inoculated to attain an initial concentration of ~10^6^ CFU/mL of the following bacteria: *E. coli* (blue bars), *L. monocytogenes* (red bars), and *S. enterica* (green bars). Strains utilized possessed antibiotic resistance markers to allow enumeration of the specific pathogens on agar plates containing antibiotics. The number of colony forming units (CFU) per mL of sample is shown for the three pathogen after each of the four treatments (glass wool, 50 µm filter, graphite felt, and continuous flow centrifugation (CFC)). Because samples were processed via the Stomacher prior to the above listed treatments, the stomached samples represent the initial reference point (the inoculum). In addition, for inclusiveness, the CFU/mL is listed for both the liquid retentate and the effluent of the CFC.

Although some antibiotic resistant colonies were seen for the most concentrated spinach samples plated on both streptomycin and kanamycin, there were no antibiotic resistant colonies at the sample dilution levels from which the CFU enumeration presented in this paper was calculated. Three independent trials were performed for the different filtration techniques (stomacher, GW, 50 μm filter, GF, and CFC) and the four food matrices (ground beef, ground pork, ground turkey, and spinach) with each individual trial consisting of replicating each 6 × 6 plate in triplicate on all three antibiotics (spectinomycin, streptomycin, and kanamycin). The number of pathogenic bacterial cells used as inoculum and the number of pathogenic bacteria in the CFC effluent were also quantitated. All of the aforementioned cell counts are reported in Figure 5.

The data was grouped by food matrix, and enumerated pathogen associated with each condition. The results of Student’s t-tests, α = 0.05, demonstrated that most of the bacteria used in the inoculum were not trapped in the food matrices or the filter materials during the processing and passed through into the filtrate as expected since the number of CFUs/mL obtained post-processing was not statistically distinct to that used in the inoculum for the GW, 50 μm filter, and the GF. However, because the CFC is a centrifugation technique and involves the concentration of samples, it was hypothesized that the CFUs would be higher post-processing than in the inoculum. The data in this study confirmed that hypothesis with all samples processed via the CFC resulting in a statistically significant increase in the number of CFU/mL compared to the inoculum, mainly due to volume reduction (to approx. 25% of the initial volume). The statistically lower level of CFUs observed in the effluent of the CFC also serves to support this conclusion. It is important to note that for the ground meat samples the sum of the cells found in the liquid retentate in the CFC bowl and the CFC effluent does not equal the total number of cells used as inoculum for the CFC (Table 1). It is likely that the remaining cells were trapped in the solid retentate that is attached to the side of the bowl after centrifugation (Figure 1A–C).

In general, the recovery of both Gram-positive (*L. monocytogenes*) and Gram-negative (*E. coli* and *S. enterica*) bacteria were similar for each separation technique tested. This suggests the utility for all of the described separation platforms when processing samples involving these and possibly other food matrices.

### 3.4. Use of GW Filtration Prior to CFC Decreased Losses of Pathogenic Bacteria Associated with CFC

As with most separation techniques, additive effects important for sample preparation may be seen when two or more techniques are used. To demonstrate this, inoculated ground beef samples were processed using CFC with and without pre-filtration via glass wool. *E. coli*, *L. monocytogenes*, and *S. enterica* process via the CFC or the GW + CFC was enumerated for both the liquid retentate of the bowl and the effluent (Table 1).

**Figure 6:**
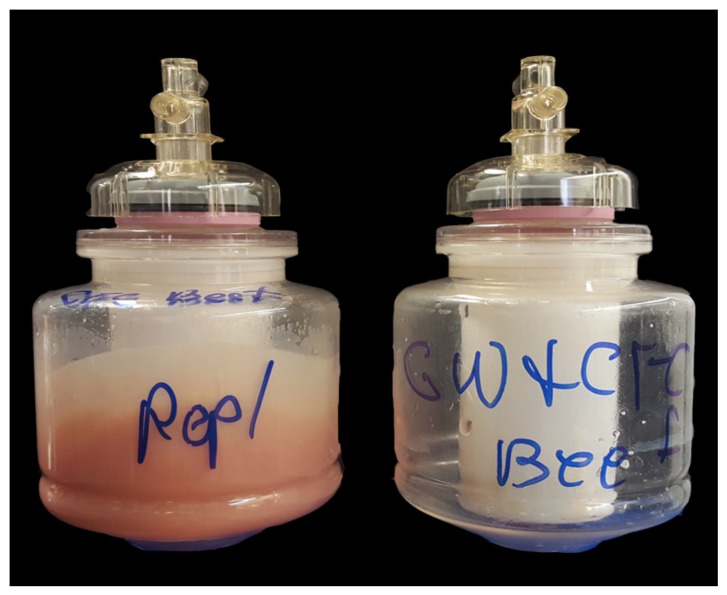
The use of glass wool (GW) prior to continuous flow centrifugation (CFC) eliminates the solid retentate resulting from CFC. Deposits that typically accumulate along the wall of the CFC bowl when ground beef was processed via the CFC were eliminated by filtering samples with the GW before CFC. The sample bowl on the left is representative of the ground beef stomached sample processed via CFC. The sample bowl on the right is representative of the CFC bowl of ground beef stomached sample filtered using GW prior to CFC.

Results demonstrated significantly increased recovery of cells in the CFC bowl for all three members of the inoculum when the GW was used in tandem with the CFC compared to the use of CFC alone. In addition, the large amount of solid retentate attached to the side of the bowl typically observed post-processing of ground beef via the CFC was not present when the GW was used prior to CFC (Figure 6).

## 4. Discussion

Sensitive techniques for the molecular detection and identification of microbes are currently available; however, food samples often require clarification before these techniques can be applied. This need for suitable sample preparation is widespread and multiple different methods may be successfully applied to food samples when paired with specific downstream detection methods. For example, PCR-based methods can be employed for the detection of pathogenic *E. coli* and *Salmonella* in beef [28]. However, it is known that certain components present in the food matrix such as blood or salts interfere with the detection of these pathogens via PCR, by either interacting with the DNA, which affects the binding/processivity of the DNA polymerase, or by scavenging cofactors necessary for the activity of the polymerase. When inhibitors are identified, sample preparation techniques with attributes that facilitate their removal can be employed to help eliminate false negatives resulting from assay failure. In general, it may save both time and resources to predetermine certain properties that a separation technique will yield post-processing, as compared to identifying compatible techniques empirically for all of the different downstream applications. Although filtration techniques such as glass wool have been shown to be functional, information concerning how they affect the sample or what materials are being removed is sparse. 

To provide a framework for the deductive selection of an effective sample preparation technique, particle size, composition and bacterial retention both pre- and post- implementation were analyzed for several sample preparation methods including continuous flow centrifugation, glass wool, graphite felt, and 50 μm polypropylene filters. Although there is concern regarding the ability of bacteria to bind to matrix components [40,41,42,43], the study herein did not attempt to incorporate a “release agent” [44,45,46,47,48] and simply added the inoculum to the matrix without an incubation step to lessen the likelihood of bacteria/ matrix interactions and avoid confounding the analysis. However, we acknowledge this concern and recognize that addition of any such agent may alter the effects of the clarification processes presented here.

Sample processing via the CFC is unique in several aspects compared to those processed via the other filtration techniques presented in this study (GW, 50 μm filter, GF). First, the CFC was the only method utilized that appeared to concentrate the sample upon processing. This concentration effect was seen with both the food matrix components (Figure 3) and the microbial cell counts for *L. monocytogenes, E. coli* O157:H7, and *S. enterica* (Figure 5) as expected, since centrifugation is often utilized as a method to concentrate bacteria or other sample components [26]. It is important to note that during sample processing, a large amount of solid retentate was deposited along the inside of the CFC bowl during the treatment of the beef, pork and turkey products but not during the treatment of the spinach (Figure 1), and that no attempt was made to reincorporate the solid retentate into the liquid retentate in the bowl. Based on the principles behind centrifugation, it is likely that the solid retentate is composed of large, dense particulate matter that was compressed along the wall of the bowl. This is consistent with the changes seen in the particle size distribution, where the volume % of larger particles was reduced in the meat samples processed via the CFC (Figure 2). Because the liquid was decanted and the pellet *not* redispersed, these particles are effectively removed during the process, an observation supported by the relative concentration data provided by the Malvern particle size analyzer software. Conversely, a decrease in volume % of larger particles was not observed with spinach, indicating a difference in density amongst the large spinach particles compared to meat particles of analogous size. Intriguingly, because the CFC does not remove much fat from the sample (and in some cases was even shown to concentrate the fat in the sample) (Figure 3), it was likely that, despite its appearance (Figure 1), the solid retentate was not simply comprised of fats. Additional studies would be necessary to define the composition of the solid retentate, which was considered superfluous information for the scope of this work.

Differences in microbial retention were also correlated with the deposition of the solid retentate in the CFC bowl. For example, in spinach most of the inoculated cells were accounted for either through the bacterial counts found in the liquid retentate in the bowl or those found in the CFC effluent. This was not the case, however, for the meat products, as ~69, 30, and 41% of the *E. coli* cells were unaccounted for post-processing of the beef, pork and turkey samples, respectively (Table 1). These cells are likely associated with the material deposited on the walls of the CFC bowl. Taken together, these data imply that spinach homogenates can be processed without a prefilter. However, a prefilter that can remove the large particles in the meat samples (100–1000 µm), such as a 50 μm filter, glass wool or graphite felt, can be used to prevent the formation of the pellet, which appears to trap some of the bacteria of interest. This was confirmed when inoculated ground beef was clarified with GW followed by CFC. Results of this experiment demonstrated that the use of these techniques in tandem increased the recovery of *L. monocytogenes, E. coli* O157:H7, and *S. enterica* in the CFC liquid retentate of the bowl from 32, 18, and 27% to 79, 48, and 62%, respectively (Table 1). This information could be of high importance when considering the CFC for clean-up depending upon the sample chosen for processing.

Material costs are another important factor to consider, especially during the commercialization of a proven detection platform. Of the techniques presented here, the graphite felt was the most expensive, with an approximate cost of $76 per filter when both the price of the graphite felt and the size utilized was considered. Although the price associated with the disposable bowls and tubing necessary for the CFC may be less than the graphite felt, there is an initial set-up cost associated with the purchase of a continuous flow centrifugation machine that should be considered. The most economical approach, of the four separation techniques presented, was the glass wool because not only was the glass wool inexpensive but it has multiple manufacturers and thus the greatest potential for price negotiation. 

## 5. Conclusions

Sample preparation for detection of microbial pathogen contamination in food is not one size fits all. The selection of an appropriate preparation technique is interdependent upon the criteria and constraints associated with the particular detection assay. Common objectives include the removal of specific components from heterogeneous sample mixtures as they may interfere with a select detection mechanism or generate potential pitfalls such as clogging or fouling of sensor surfaces. Our data indicates that each technique evaluated has a unique ability to remove particles within different size classes and macronutrient components from complex mixtures, whereas the sequential application of the different techniques can potentially amplify one’s ability to eliminate unwanted components. This study also verified that the techniques do not cause the unintended consequence of removing target microorganisms from the sample, which could produce false negative responses from down-stream detection applications. While this study provides a framework for the identification of methods with superior qualities for the removal of unwanted substances, the decision regarding the most effective application ultimately relies on careful consideration of the requirements of the downstream detection techniques.

## Figures and Tables

**Table 1:** Cell count of the different fractions resulting from sample processing via CFC.

Condition	Food Matrix	Pathogen	% Retentate	% Effluent	% Unaccounted
CFC ^1^	Ground Pork	*E. coli* O157:H7	49.2%	21.1%	29.7%
		*L. monocytogenes*	32.5%	24.6%	42.9%
		*S.* Minnesota	41.4%	33.9%	24.7%
CFC ^1^	Ground Turkey	*E. coli* O157:H7	34.9%	24.1%	41.0%
		*L. monocytogenes*	45.4%	29.9%	24.7%
		*S.* Minnesota	59.6%	40.4%	0.0%
CFC ^1^	Raw Spinach	*E. coli* O157:H7	75.1%	12.4%	12.5%
		*L. monocytogenes*	69.8%	23.0%	7.2%
		*S.* Minnesota	74.9%	25.1%	0.0%
CFC ^1^	Ground Beef	*E. coli* O157:H7	18.4%	12.2%	69.4%
		*L. monocytogenes*	32.4%	2.7%	64.9%
		*S.* Minnesota	27.4%	21.6%	51.0%
GW + CFC ^2^	Ground Beef	*E. coli* O157:H7	48.0%	11.3%	40.7%
		*L. monocytogenes*	79.0%	21.0%	0.0%
		*S.* Minnesota	61.5%	18.1%	20.4%

^1^ Samples processed using continuous flow centrifugation (CFC); ^2^ Samples processed using glass wool prior to CFC.
